# Tissue-agnostic biomarkers in solid tumors: current approvals and emerging candidates

**DOI:** 10.1007/s10555-025-10274-2

**Published:** 2025-06-27

**Authors:** Jinah Kim, Hye Sung Kim, Myungwoo Nam, Young Kwang Chae

**Affiliations:** 1 Division of Hematology and Oncology, Department of Medicine, Larner College of Medicine at University of Vermont, Burlington, VT USA; 2 Department of Medicine, Lewis Katz School of Medicine at Temple University, Temple University Hospital, Philadelphia, PA USA; 3 Division of Hematology and Oncology, Department of Medicine, SUNY Upstate Medical University, Syracuse, NY USA; 4 Division of Hematology and Oncology, Department of Medicine, Northwestern University Feinberg School of Medicine, Chicago, IL USA

**Keywords:** Tissue-agnostic biomarker, Pan-tumor biomarker, Precision oncology, Targeted therapy, Immunotherapy, Tumor-infiltrating lymphocytes

## Abstract

The landscape of cancer treatment has shifted from histology-specific to tissue-agnostic approaches, targeting molecular alterations regardless of tumor origin. Currently, six pan-cancer biomarkers—NTRK, BRAF V600E, RET, HER2-positive, MSI-high, and TMB-high—along with nine molecularly targeted therapies have expanded treatment options across diverse malignancies. This review examines each biomarker’s molecular basis, prevalence across tumor types, and corresponding FDA-approved therapies. Additionally, emerging candidates—including FGFR, ALK, MET, ROS1, NRG1, PIK3CA, AKT, KRAS G12C, HER2 mutations, HER2-low/ultralow, B7-H3, and tumor-infiltrating lymphocytes (TILs)—are explored. While these biomarkers represent a paradigm shift in oncology, their integration into clinical practice requires overcoming challenges related to tumor heterogeneity and lineage-specific molecular dependencies. Future research should focus on identifying novel biomarkers, optimizing treatment strategies through multiomic analyses, and leveraging innovative clinical trial designs to advance precision oncology. In particular, further investigation into TILs as a predictive biomarker for immunotherapy is warranted, given their distinct immunophenotypic features and prognostic significance in shaping treatment responses across cancer types.

**Graphical Abstract:**

Abbreviations: ADC, antibody–drug conjugate; AKT, protein kinase B; ALK, anaplastic lymphoma kinase; APC, antigen-presenting cell; B7-H3, B7 homolog 3; BRAF, B-raf proto-oncogene; CTLA-4: cytotoxic T-lymphocyte-associated protein 4; DNA, deoxyribonucleic acid; ERK, extracellular signal-regulated kinase; FGFR, fibroblast growth factor receptor; GzmB, granzyme B; HER, human epidermal growth factor receptor; IFNγ, interferon-gamma; KRAS, Kirsten rat sarcoma viral oncogene homolog; MEK, mitogen-activated protein kinase kinase; MET, mesenchymal-epithelial transition factor; MSI, microsatellite instability; mTOR, mechanistic target of rapamycin; NRG1, neuregulin 1; NTRK, neurotrophic tyrosine receptor kinases; PD-1, programmed death receptor-1; PFN, perforin; PI3K, phosphoinositide 3-kinase; PIK3CA, phosphatidylinositol-4,5-bisphosphate 3-kinase catalytic subunit alpha; RET, rearranged during transfection; ROS1, proto-oncogene receptor tyrosine kinase 1; T-DXd, fam-trastuzumab deruxtecan-nxki; TIL, tumor-infiltrating lymphocytes; TKI, tyrosine kinase inhibitor; TMB, tumor mutation burden; TNFα, tumor necrosis factor-alpha.

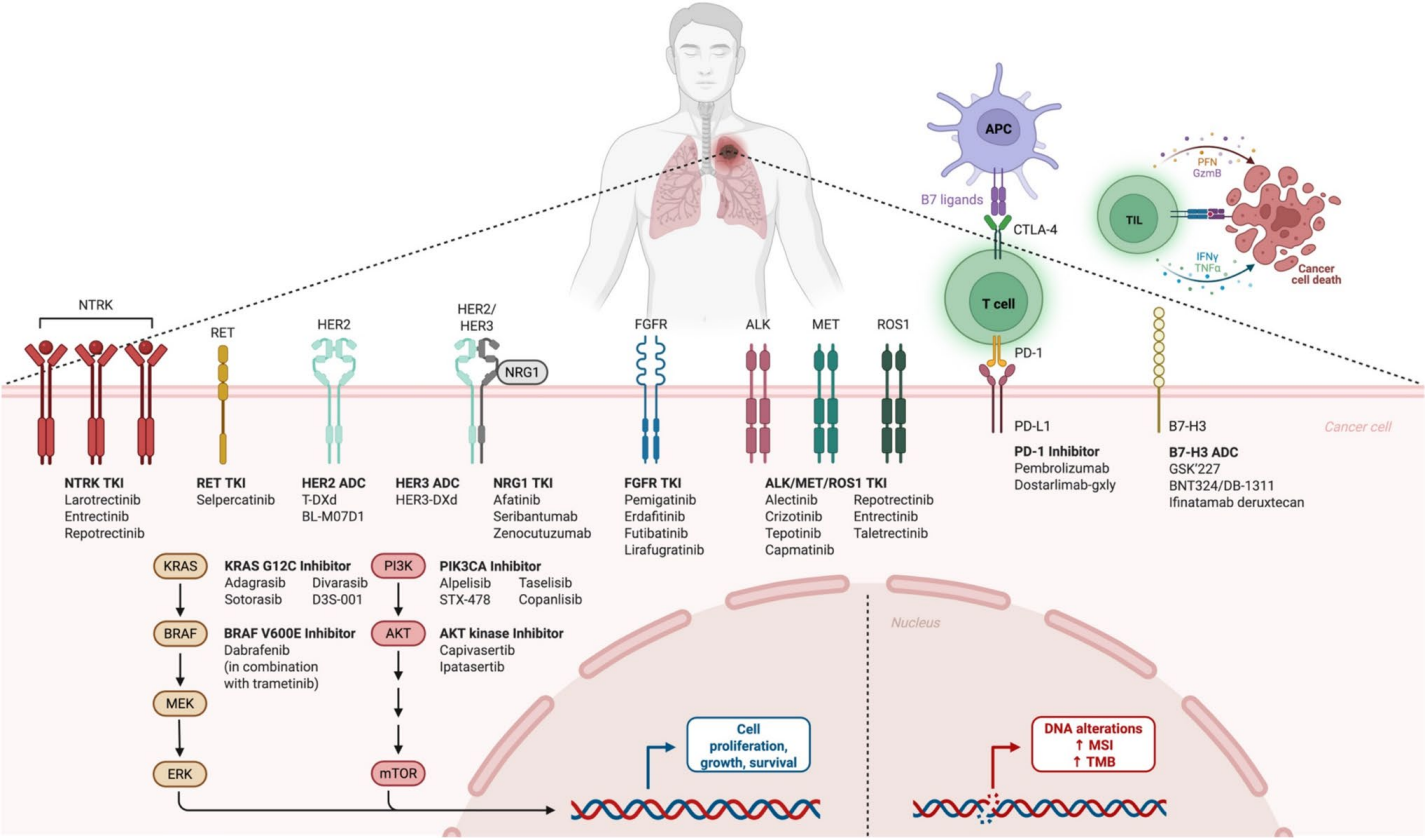

## Background

In April 2024, the US Food and Drug Administration (FDA) granted accelerated approval to fam-trastuzumab deruxtecan-nxki for HER2-positive solid tumors, establishing HER2 as the fourth FDA-approved tissue-agnostic biomarker for targeted therapy and the sixth when including both targeted therapy and immunotherapy. This milestone underscores the ongoing shift in oncology from traditional histology-based treatments to molecularly targeted approaches that transcend tumor type.

The precision medicine paradigm enables more tailored interventions, broadening treatment options across diverse malignancies. The clinical impact of this shift is supported by a meta-analysis of 570 phase II clinical trials, which demonstrated that biomarker-driven precision oncology significantly improves outcomes, yielding higher response rates (31% vs. 10.5%), longer median progression-free survival (mPFS) (5.9 vs. 2.7 months), and extended overall survival (13.7 vs. 8.9 months) compared to non-biomarker-driven strategies [1].

Advancements in technology, such as the widespread adoption of liquid biopsies, have made genetic testing more accessible and integral to developing treatments precisely tailored to the genetic profiles of individual tumors. Additionally, histology-agnostic basket trials (e.g., NCI-MATCH [2], ComboMATCH [3], DETERMINE [4]) are streamlining the evaluation of novel therapies, expediting their integration into clinical practice [5–7].

This review synthesizes the evolving landscape of tissue-agnostic biomarkers, examining FDA-approved therapies, ongoing pan-tumor clinical trials, and emerging biomarker candidates. By exploring advances in both targeted therapy and immunotherapy, we highlight how these developments are redefining oncology, setting new standards of care, and improving patient outcomes across multiple cancer types.

## Current FDA-approved tissue-agnostic biomarkers (Table 1)

**Table 1:** FDA-approved tissue-agnostic biomarkers in oncology

Biomarker	Drug	Mechanism of action	Date of approval	Tissue-agnostic trial		Phase	*N*	ORR (95% CI)	mDOR (95% CI)	mPFS (95% CI)	mOS (95% CI)	Data cutoff date
Targeted therapy												
NTRK fusion	Larotrectinib	TRK inhibitor	11/2018	Pooled cohort			55	75% (61–85)	NR	NR	NA	2/2017
							180	57% (50–65)	43.3 (29.2–NE)	24.6 (11.3–34.5)	48.7 (38.5–NE)	7/2022
				LOXO-TRK-14001 (NCT02122913)		I						
				SCOUT (NCT02637687)		I/II						
				NAVIGATE (NCT02576431)		II						
	Entrectinib		8/2019	Pooled cohort			54	57% (43.2–70.8)	10 (7.1–NE)	NA	NA	5/2018
							150	61.3% (53. –69.2)	20 (13.2–31.1)	13.8 (10.1–20.0)	37.1 (27.2–NE)	8/2021
				ALKA-372–001 (NCT02097810)		I						
				STARTRK-1 (NCT02097810)		I						
				STARTRK-2 (NCT02568267)		II						
	Repotrectinib		6/2024	TRIDENT-1 (NCT03093116)		I/II	88	TKI-naïve: 58% (41–73) TKI-pretreated: 50% (35–65)	TKI-naïve: NE TKI-pretreated: 9.9 (7.4–13.0)	NA	NA	12/2022
BRAF V600E mutation	Dabrafenib (in combination with trametinib)	RAF inhibitor	6/2022	Pooled cohort			131	41% (33–50)	NA	NA	NA	NA
				ROAR/BRF117019 (NCT02034110)		II						
				NCI-MATCH subprotocol H (NCT02465060)		II						
				CTMT212X2101 (NCT02124772)		I/IIa	36	25% (12.1–42.2)	33.6 (11.2–NR)	36.9 (36.0–NR)	NA	NA
RET fusion	Selpercatinib	RET kinase inhibitor	1/2023	LIBRETTO-001 (NCT03157128)		I/II	41	44% (28–60)	24.5 (9.2–NE)	13.2 (7.4–26.2)	NA	9/2022
							52	44.2% (30.5–58.7)	37.2 (13.3–NE)	13.2 (5.6–26.2)	NA	1/2023
HER2-positive (IHC 3 +)	Fam-trastuzumab deuxtecan-nxki	Anti-HER2 ADC	4/2024	DESTINY-PanTumor02 (NCT04482309)		II	111	51.4% (41.7–61.0)	19.4 (1.3–27.9 +)	NA	NA	NA
				DESTINY-Lung01 (NCT03505710)		II	17	52.9% (27.8–77.0)	6.9 (4.0–11.7 +)	NA	NA	NA
				DESTINY-CRC02 (NCT04744831)		II	64	46.9% (34.3–59.8)	5.5 (1.3 + to 9.7 +)	NA	NA	NA
Immunotherapy												
MSI-H/dMMR	Pembrolizumab	Anti-PD-1 monoclonal antibody	5/2017	Pooled cohort			149	39.6% (31.7–47.9)	NR	NA	NA	NA
				KEYNOTE-016 (NCT01876511)		II						
				KEYNOTE-164 (NCT02460198)		II						
				KEYNOTE-012 (NCT01848834)		I						
				KEYNOTE-028 (NCT02054806)		I						
				KEYNOTE-158 (NCT02628067)		II						
			3/2023	Pooled cohort			504	33.3% (29.2–37.6)	63.2 (NA)	NA	NA	NA
				KEYNOTE-158 (NCT02628067)		II						
				KEYNOTE-164 (NCT02460198)		II						
				KEYNOTE-051 (NCT02332668)		I/II						
	Dostarlimab-gxly	Anti-PD-1 monoclonal antibody	8/2021	GARNET (NCT02715284)		I	209	41.6% (34.9–48.6)	34.7 (NA)	NA	NA	NA
							327	44% (38.6–49.6)	NR	6.9 (4.2–13.6)	NR	11/2021
TMB-H	Pembrolizumab	Anti-PD-1 monoclonal antibody	6/2020	KEYNOTE-158 (NCT02628067)		II	102	29.4 (20.8–39.3)	NR	2.1 (2.1–4.1)	11.7 (9.1–19.1)	6/2019

*ADC*, antibody–drug conjugate; *BRAF*, B-raf proto-oncogene; *CI*, confidence interval; *dMMR*, mismatch-repair deficiency; *FDA*, Food and Drug Administration; *HER*, human epidermal growth factor receptor; *IHC*, immunohistochemistry; *mDOR*, *median duration of response; mOS*, median overall survival; *mPFS*, median progression-free survival; *MSI-H*, microsatellite instability-high; *NA*, not available; *NE*, not estimable; *NR*, not reached; *NTRK*, neurotrophic tyrosine receptor kinases; *ORR*, objective response rate; *PD-1*, programmed death receptor-1; *RAF*, rapidly accelerated fibrosarcoma; *RET*, rearranged during transfection; *TKI*, tyrosine kinase inhibitor; *TMB-H*, tumor mutation burden-high; *TRK*, tropomyosin receptor kinase

### Biomarkers for targeted therapy

#### Neurotrophic tyrosine receptor kinases (NTRK) fusion

NTRK gene fusion became the second tissue-agnostic biomarker following the FDA’s approval of larotrectinib in November 2018 for unresectable or metastatic solid malignancies without alternative treatment options.

The NTRK family includes three genes, NTRK1, NTRK2, and NTRK3, which encode the receptor tyrosine kinases TRKA, TRKB, and TRKC, respectively. The NTRK signaling pathway is crucial for neural cell development, and alterations in NTRK genes can lead to oncogenic activation [8]. Fusion of the NTRK kinase domain with various partner genes produces a constitutively activated chimeric protein, driving tumorigenesis independently of ligand binding [9]. NTRK gene fusions occur rare overall (< 1%) but highly prevalent (> 90%) in mammary analogue secretory carcinoma and fibrosarcoma. Among common tumors, NTRK fusions are found in approximately 26% of papillary thyroid cancers, 0.7–1.5% of colorectal cancers (CRCs), and 0.2–0.3% of lung cancers [9].

Three TRK inhibitors, larotrectinib, entrectinib, and repotrectinib, are FDA-approved for unresectable or metastatic solid tumors with NTRK fusion that have progressed on prior treatment or have no alternate satisfactory options. Larotrectinib received accelerated approval based on data from 55 patients in three single-arm clinical trials: phase I LOXO-TRK-14001 (NCT02122913), phase I/II SCOUT (NCT02637687), and phase II NAVIGATE (NCT02576431), showing an objective response rate (ORR) of 75% (95% CI: 61–85) [10]. Updated results from180 patients demonstrated an ORR of 57% (95% CI: 50–65), a median duration of response (mDOR) of 43.3 months (95% CI: 29.2–not estimable [NE]), a mPFS of 24.6 months (95% CI: 11.3–34.5), and a median overall survival (mOS) of 48.7 months (95% CI: 38.5–NE) [11].

Entrectinib received accelerated approval in August 2019, based on three clinical trials: phase I ALKA-372–001 (EudraCT 2012–000148-88), phase I STARTRK-1 (NCT02097810), and phase II STARTRK-2 (NCT02568267). Approval was based on 54 patients, showing an ORR of 57.0% (95% CI: 43.2–70.8) and mDOR of 10 months (95% CI: 7.1–NE) [12]. Updated results from 150 patients reported an ORR of 61.3% (95% CI: 53.1–69.2), mDOR of 20.0 months (95% CI: 13.2–31.1), mPFS of 13.8 months (95% CI: 10.1–20.0), and mOS of 37.1 months (95% CI: 27.2–NE) [13]. Additionally, entrectinib has tissue-specific FDA approval for non-small cell lung cancer (NSCLC).

In June 2024, the FDA granted accelerated approval to repotrectinib for patients ≥ 12 years with locally advanced or metastatic NTRK fusion-positive solid tumors, based on the TRIDENT-1 trial (NCT03093116) which reported a confirmed ORR of 58% (95% CI: 41–73) in tyrosine kinase inhibitor (TKI)-naïve patients and 50% (95% CI: 35–65) in TKI-pretreated patients, with mDOR of NE and 9.9 months (95% CI: 7.4–13.0), respectively [14].

#### B-raf proto-oncogene (BRAF) V600E mutation

In June 2022, the FDA granted accelerated approval to the combination of dabrafenib and trametinib for unresectable or metastatic solid tumors with BRAF V600E mutation, establishing BRAF V600E as a pan-tumor biomarker.

The BRAF gene, located on chromosome 7, encodes the BRAF protein, part of the mitogen-activated protein kinase (MAPK)/extracellular signal-regulated kinase (ERK) pathway, also known as the Ras-Raf-MEK-ERK pathway. Activated ERK regulates transcription factors leading to cell proliferation and growth [15]. BRAF mutations are reported in about 7% of solid tumors, with V600E being the most common. This mutation is prevalent in papillary thyroid cancer, melanoma, and CRC [16].

Dabrafenib and trametinib are kinase inhibitors that block BRAF and MEK, respectively. Their combination effectively inhibits the MAPK/ERK signaling pathway at two different points, disrupting tumor cell proliferation [15]. The combination of dabrafenib and trametinib was approved based on the results of three multicenter clinical trials: phase II ROAR/BRF117019 (NCT02034110), phase II NCI-MATCH subprotocol H (NCT02465060, EAY131-H), and phase I/IIa CTMT212X2101 (NCT02124772). Among 131 adult patients, the ORR was 41% (95% CI: 33–50), while in 36 pediatric patients, the ORR was 25% (95% CI: 12–42).

The most recent updates from the ROAR/BRF117019 trial reported data from 108 adults, with ORRs varying by tumor type [17]. The ORR ranged from 33% in high-grade glioma (HGG) (*n* = 45) to 89% in hairy cell leukemia (*n* = 55). The mDOR ranged from 7.7 months in adenocarcinoma of the small intestine (*n* = 3) to 31.2 months in HGG. The mPFS ranged from 5.5 months in HGG to 9.5 months in low-grade glioma (LGG) (*n* = 13), while the mOS ranged from 13.5 months in biliary tract cancer (*n* = 43) to 33.9 months in multiple myeloma (*n* = 10). The NCI-MATCH subprotocol H, which included 29 adult patients, showed an ORR of 37.9% (95% CI: 22.9–54.9), mDOR of 25.1 months (95% CI: 12.8–NR), mPFS of 11.4 months (95% CI: 8.4–16.3), and mOS of 28.6 months [18]. Updates from the CTMT212X2101 in 36 pediatric patients with LGG showed an ORR of 25% (95% CI: 12.1–42.2), mDOR of 33.6 months (11.2–NR), and mPFS of 36.9 months (95% CI: 36–NR) [19]. The dabrafenib-trametinib combination has cancer-specific FDA approval for melanoma, NSCLC, and anaplastic thyroid cancer.

#### Rearranged during transfection (RET) fusion

Selpercatinib received accelerated approval in September 2022 for adults with locally advanced or metastatic RET fusion-positive solid tumors that had progressed on prior systemic treatment or lacked satisfactory treatment alternatives. With this approval, RET fusion was recognized as a tissue-agnostic biomarker by the FDA.

RET is a receptor tyrosine kinase that requires glial cell-derived neurotrophic factor (GDNF) family ligands and GDNF family receptor-α (GFRα) co-receptors for activation. The GDNF ligand-GFRα co-receptor-RET complex activates RAS, MAPK, ERK, PI3K, and AKT signaling pathways, promoting tumor cell proliferation, migration, and differentiation. RET fusions and point mutations lead to constitutive ligand-independent activation, driving cancer development. RET fusion has been detected in approximately 10–20% of papillary thyroid cancers, 1–2% of NSCLCs, and lower frequencies in other tumors [20, 21].

Selpercatinib, a highly selective RET kinase inhibitor that functions through an ATP-competitive mechanism, initially received FDA approval for NSCLC and medullary thyroid cancer [22, 23]. Its tissue-agnostic approval was based on the phase I/II LIBRETTO-001 trial (NCT03157128), in which an initial cohort of 41 patients with locally advanced or metastatic RET fusion-positive solid tumors (excluding NSCLC and thyroid cancer) demonstrated an ORR of 43.9% (95% CI: 28.5–60.3) and a mDOR of 24.5 months (95% CI: 9.2–NE) [24]. An updated analysis in 52 patients showed a consistent ORR of 44.2% (95% CI: 30.5–58.7), with an extended mDOR of 37.2 months (95% CI: 13.3–NR) [25].

Pralsetinib, another RET tyrosine kinase inhibitor with tissue-specific approvals for NSCLC and medullary thyroid cancer, was evaluated in the phase I/II pan-tumor ARROW trial (NCT03037385) [26, 27]. In 23 patients with RET-altered solid tumors excluding NSCLC and thyroid cancer, pralsetinib achieved an ORR of 57% (95% CI: 35–77), mDOR of 11.7 months (95% CI: 5.5–19.0), mPFS of 7 months (95% CI: 5.1–13.6), and mOS of 13.6 months (95% CI: 7.5–NR) [28].

#### Human epidermal growth factor receptor 2 (HER2) overexpression

In April 2024, the FDA granted accelerated approval to fam-trastuzumab deruxtecan-nxki (T-DXd) for adults with unresectable or metastatic immunohistochemistry (IHC) 3 + HER2-positive solid tumors who have received prior systemic treatment and have no satisfactory alternative options.

HER2, also known as ERBB-2, is a member of the EGFR family, which includes HER1 (EGFR, ERBB1), HER2 (ERBB2), HER3 (ERBB3), and HER4 (ERBB4). HER2 overexpression promotes heterodimerization with other EGFR family members, leading to the autophosphorylation of tyrosine residues in the cytoplasmic domain of the heterodimer. This activation triggers growth factor signaling pathways such as RAS-RAF-MEK-ERK and PI3K-AKT-mTOR, promoting cell proliferation and survival [29].

T-DXd is a HER2-directed antibody–drug conjugate (ADC). It is comprised of trastuzumab, a monoclonal antibody targeting HER2, and a payload, deruxtecan (DXd), which is a DNA topoisomerase 1 inhibitor (a camptothecin analog) with cytotoxic activity connected by a cleavable linker that allows intracellular drug release. Upon binding to HER2 on tumor cells, T-DXd undergoes internalization, followed by lysosomal cleavage of the linker. The released DXd payload inhibits DNA replication, leading to cell cycle arrest and apoptosis [30].

The tissue-agnostic approval of T-DXd was based on data from three phase II DESTINY trials. Results from 111 patients with HER2 IHC 3 + across 20 cancer types in DESTINY-PanTumor02 demonstrated an ORR of 51.4% (95% CI: 41.7–61.0) and mDOR of 19.4 months (range: 1.3–27.9 +). Among the seven cohorts, the ORR was notably high in endometrial (ORR 57.5%, 95% CI: 40.9–73.0) and cervical (ORR 50.0%, 95% CI: 33.8–66.2) cohorts while lower in the biliary tract (ORR 22.0%, 95% CI: 10.6–37.6) and pancreatic (ORR 4.0%, 95% CI: 0.1–20.4) cohort [31]. In the DESTINY-Lung01 trial, the efficacy and safety of T-DXd were evaluated in patients with HER2 mutated or overexpressed NSCLC. Results from 17 patients with HER2 IHC 3 + who received a 5.4 mg/kg dose showed an ORR of 52.9% (95% CI: 27.8–77.0), mPFS of 7.5 months (95% CI: 3.1–11.0), and mOS of 12.5 months (95% CI: 9.3–NE) [32]. Data from 64 patients with HER2 IHC 3 + metastatic CRC in DESTINY-CRC02 demonstrated an ORR of 46.9% (95% CI: 34.3–59.8) and mDOR of 5.5 months (95% CI: 4.2–8.1) [33].

Regarding tissue-specific indications, T-DXd is approved for the treatment of the unresectable or metastatic HER2-positive (IHC 3 + or *in situ* hybridization (ISH) +) breast cancer in patients who have received prior anti-HER2 therapy, hormone receptor-positive HER2-low (IHC 1 + or IHC 2 +/ISH-) or HER2-ultralow (IHC 0 with membrane staining) breast cancer, HER2-low breast cancer following prior chemotherapy or endocrine therapy, HER2-mutated NSCLC under accelerated approval, and HER2-positive (IHC 3 + or IHC 2 +/ISH +) gastric or gastroesophageal junction (GEJ) adenocarcinoma after trastuzumab-based treatment.

Additionally, zanidatamab-hrii, a bispecific HER2-directed antibody, is emerging as a novel targeted therapy for HER2-expressing solid tumors and is currently being investigated in multiple clinical trials. On November 20, 2024, the FDA granted accelerated approval to zanidatamab-hrii for the treatment of previously treated, unresectable or metastatic HER2-positive (IHC 3 +) biliary tract cancer. This approval suggests the expanding role of HER2-targeted therapies and reinforces the importance of HER2 expression as a clinically relevant biomarker across multiple malignancies.

### Biomarkers for immunotherapy

#### Microsatellite instability (MSI)

The FDA’s approval of pembrolizumab for unresectable or metastatic solid tumors with high microsatellite instability (MSI-H) or mismatch repair deficiency (dMMR) in adults and children represented the first biomarker-driven pan-tumor approval. This decision was based on the understanding that MSI-H and dMMR are key molecular features that impact tumor biology.

Microsatellites are short tandem repeats of one to six nucleotide sequences scattered throughout the human genome [34]. Due to DNA polymerase slippage, these regions are prone to replication errors, which are typically corrected by mismatch-repair proteins, such as MLH1, MSH2, MSH6, and PMS2 [35]. However, when MMR function is lost, these errors accumulate, resulting in MSI-H, which is associated with high tumor mutational burden (TMB), enhanced neoantigen formation, and increased sensitivity to immune checkpoint inhibitors (ICIs) [36].

Given the strong biological rationale for targeting MSI-H tumors, early research primarily focused on endometrial and gastrointestinal cancers, where MSI-H is most prevalent [37]. This led to the National Comprehensive Cancer Network (NCCN) recommending universal MSI/MMR testing for newly diagnosed endometrial, colorectal, small bowel, gastric, and esophageal cancers to guide treatment decisions. One such treatment, pembrolizumab, is a humanized monoclonal IgG4 kappa antibody that targets programmed death receptor-1 (PD-1) on lymphocytes. Many tumors express programmed death receptor ligand-1 (PD-L1) (range: 14–100%), which interacts with PD-L1 to suppress T-cell function [38]. By inhibiting this interaction, pembrolizumab restores T-cell-mediated tumor cell killing, making it an effective treatment for MSI-H/dMMR malignancies.

In May 2017, the FDA granted pembrolizumab accelerated approval based on five uncontrolled single-arm clinical trials (KEYNOTE-016, KEYNOTE-164, KEYNOTE-012, KEYNOTE-028, and KEYNOTE-158) involving 149 patients across 15 types of unresectable or metastatic MSI-H/dMMR solid tumors that had progressed after prior treatment and had no satisfactory alternatives. The most common MSI-H tumors included colorectal, endometrial, and gastrointestinal cancers, with an ORR of 39.6% (95% CI: 31.7–47.9) [39]. This was followed by full approval in March 2023, supported by data from the phase II KEYNOTE-158, KEYNOTE-164, and KEYNOTE-051 trials, including 504 patients across more than 30 cancer types. In a pooled analysis with a median follow-up of 20.1 months (range: 0.1–71.4), pembrolizumab achieved an ORR of 33.3% (95% CI: 29.2–37.6) [40–42].

In August 2021, dostarlimab-gxly, another PD-1 monoclonal antibody, was approved for adult patients with dMMR recurrent or advanced solid tumors that had progressed following prior treatment and had no satisfactory alternatives. This approval was based on the GARNET trial (NCT02715284), an ongoing phase I trial involving 209 patients with dMMR solid tumors. The trial reported an ORR of 41.6% (95% CI: 34.9–48.6), with 19 patients achieving a complete response (CR) and 68 achieving a partial response (PR). The mDOR was 34.7 months (range: 2.6–35.8 +) [43].

#### Tumor mutation burden (TMB)

TMB is the third tissue-agnostic biomarker and the second biomarker for pembrolizumab. In June 2020, the FDA granted accelerated approval to pembrolizumab for adult and pediatric patients with unresectable or metastatic tumor mutational burden-high (TMB-H) [≥ 10 mutations/megabase] solid tumors that had progressed after prior treatment and had no satisfactory alternatives.

TMB is a continuous, quantitative variable that is associated with the likelihood of neoantigen generation in tumor cells. Defined as the total number of nonsynonymous mutations per megabase of the tumor genome, TMB serves as an indirect measure of a tumor’s potential to produce immunogenic neopeptides displayed on major histocompatibility complexes. While not all mutations generate neoantigens, a higher mutation load correlates with an increased probability of immunogenic neoantigen formation and response to immunotherapy [44, 45].

The KEYNOTE-158 trial (NCT02628067), which led to pembrolizumab’s accelerated approval, identified 102 patients (13%) with TMB-H tumors, with responses observed across eight tumor types. The ORR was 29.4% (95% CI: 21–39), including 4 CRs and 26 PRs. The mDOR was not reached, with 66% of responders maintaining a response for 24 months or longer at the time of data cutoff [46, 47].

## Emerging candidates of tissue-agnostic biomarkers (Table 2)

**Table 2:** Emerging tissue-agnostic biomarkers in oncology

Biomarker	Drug	Mechanism of action	Tissue-agnostic trial	Phase	Results	Tissue-specific indication	Date of approval
**Targeted**							
FGFR amplification, mutation, and rearrangement	Pemigatinib	FGFR1/2/3 inhibitor	NCT06022289	II	NA	FGFR2-positive CCA FGFR1-positive myeloid/lymphoid neoplasm	4/2020 8/2022
			NCT06302621 (with Afatinib)	I	NA		
	Erdafitinib	FGFR1-4 inhibitor	RAGNAR (NCT04083976)	II	*n* = 217, ORR 30%, mPFS 4.2, mOS 10.7	FGFR3-positive urothelial carcinoma	1/2024
			NCI-MATCH Subprotocol K2 (NCT06351371)	II	*n* = 25, ORR 16%, mPFS 3.6, mOS 11.0		
	Futibatinib		NCT04189445	II	*n* = 115, NA	FGFR2-positive CCA	9/2022
	Lirafugratinib	FGFR2 inhibitor	ReFocus (NCT04526106)	I/II	TKI-naïve CCA: *n* = 25, ORR 52% Prior-TKI CCA: *n* = 50, ORR 14% TKI-naïve non-CCA: *n* = 45, ORR 37.8%	FGFR2-positive CCA and other solid tumors	12/2024^a^
ALK/MET/ROS1 alterations	Alectinib	ALK inhibitor	iMATRIX (NCT04774718)	I/II	Pediatric: *n* = 8, ORR 57.1%	ALK-positive NSCLC ALK-positive NSCLC	11/2017 4/2024
			DETERMINE Arm 01 (NCT05770037)	III	NA		
	Crizotinib	ALK/c-MET/ROS1 inhibitor	AcSé (NCT02034981)	II	ALK tlc ALCL: *n* = 22, ORR 54% ALK tlc/ROS1 tlc IMT: *n* = 7, ORR 28% MET mut: *n* = 27, ORR 22% MET amp NSCLC: *n* = 25, ORR 24% MET amp esogastric: *n* = 8, ORR 37% ROS1 tlc: *n* = 37, ORR 54%	ALK-positive NSCLC ROS1-positive NSCLC MET exon 14-positive NSCLC ALK-positive ALCL ALK-positive IMT	11/2013 3/2016 5/2018^a^ 1/2021 7/2022
			NCI-MATCH Subprotocol F (NCT04439266)	II	ALK + : *n* = 5, ORR 50%, mPFS 3.8, mOS 4.3		
			NCI-MATCH Subprotocol G (NCT04439253)	II	ROS1 + : *n* = 4, ORR 25%, mPFS 4.3, mOS 6.2		
			NCI-MATCH Subprotocol C1 (NCT06357975)	II	METamp: *n* = 28, ORR 14%, mPFS 3.4, mOS 7.1		
			NCI-MATCH Subprotocol C2 (NCT06360575)	II	METex14 mut/del: *n* = 14, ORR 14%, mPFS 2.0		
	Tepotinib	MET inhibitor	KCSG AL19-17 (NCT04647838)	II	*n* = 35, ORR 57.6%, mPFS 8, mOS 14	METex14-positive NSCLC	2/2024
			NCT01014936	I	IHC3 + :* n* = 16, ORR 12.5% IHC ≤ 2: *n* = 73, ORR 0% Not amplified: *n* = 71, ORR 2.8% Amplified: *n* = 9, ORR 0%		
	Capmatinib		NCT01324479	I	n = 38, ORR 0%	METex14-positive NSCLC	8/2022
	Repotrectinib	ROS1 inhibitor	CARE (NCT04094610)	I/II	*n* = 8, ORR 37.5%	ROS1-positive NSCLC NTRK fusion-positive solid tumor	11/2023 6/2024
	Entrectinib		DETERMINE Arm 03	II/III	NA	NTRK fusion-positive solid tumor ROS1-positive NSCLC	8/2019
	Taletrectinib		TRUST-II (NCT04919811)	II	NA	ROS1-positive NSCLC	6/2025
NRG1 fusion	Afatinib	EGFR inhibitor	NCT04410653	II	*n* = 3, NA	EGFR-positive NSCLC	1/2018
	Seribantumab	Anti-HER3 monoclonal antibody	CRESTONE (NCT04383210)	II	*n* = 22, ORR 36%, DOR 1.4–17.2	NRG1 fusion-positive solid tumor	5/2022^b^
	Zenocutuzumab	Anti-HER2/HER3 bispecific antibody	eNRGy (NCT02912949)	I/II	NSCLC: *n* = 64, ORR 33%, mDOR 7.4 PDAC: *n* = 30, ORR 40%, DOR 3.7–16.6	NRG1 fusion-positive NSCLC NRG1 fusion-positive PDAC	12/2024
PIK3CA mutation	Alpelisib	PI3Kα inhibitor	NCT01219699	I	*n* = 134, ORR 6%	HR-positive, HER2-negative, PIK3CA-altered breast cancer	5/2019
	STX-478		NCT05768139	I/II	*n* = 43, ORR 21%		
	Taselisib		NCI-MATCH Subprotocol I (NCT04439175)	II	*n* = 61, ORR 0%, mPFS 3.1		
	Copanlisib		NCI-MATCH Subprotocol Z1F (NCT05490771)	II	*n* = 25, ORR 16%, mPFS 3.4, mOS 5.9		
AKT mutation	Capivasertib	AKT inhibitor	NCI-MATCH Subprotocol Y (NCT04439123)	II	*n* = 35, ORR 28.6%, mPFS 5.5	HR-positive, HER2-negative, PIK3CA/AKT1/PTEN-altered breast cancer	12/2023
	Ipatasertib		NCI-MATCH Subprotocol Z1K (NCT06400251)	II	*n* = 32, ORR 22%, mDOR 9.9		
KRAS G12C mutation	Adagrasib	KRAS G12C inhibitor	KRYSTAL-1 (NCT03785249)	II	*n* = 63, ORR 35%. mDOR 5.3, mPFS 7.4	KRAS G12C-positive NSCLC KRAS G12C-positive CRC (adagrasib + cetuximab)	12/2022 6/2024
	Sotorasib		NCT04185883	I	NA	KRAS G12C-positive NSCLC KRAS G12C-positive CRC (sotorasib + panitumumab)	5/2021 1/2025
HER2 mutation	Fam-trastuzumab deruxtecan-nxki	Anti-HER2 ADC	Destiny-Pantumor01 (NCT04639219)	II	*n* = 102, ORR 29.4%	HER2-mutated NSCLC	8/2022
HER2-low (IHC 1 + or IHC 2 +/ISH −) or ultralow (IHC 0 with membrane staining)	HER3-DXd	Anti-HER3 ADC	HERTHENA-PanTumor01 (NCT06172478)	I	NA		
	BL-M07D1	Trastuzumab-based anti-HER2 ADC	NCT05470348	I	Breast: *n* = 38, ORR 50%	HER2-low breast cancer (T-DXd) HR-positive, HER2-low or HER2-ultralow breast cancer (T-DXd)	8/2022 1/2025
B7-H3	GSK5764227 (GSK’227)	Anti-B7-H3 ADC	ARTEMIS-001 (NCT05276609)	I	ES-SCLC/8 mg/kg: *n* = 31, ORR 58.1%, mDOR 4.3, mPFS 5.6, mOS NR ES-SCLC/10 mg/kg: *n* = 21, ORR 57.1%, mDOR NA, mPFS NA, mOS NR	B7-H3-positive ES-SCLC	8/2024^a^
			ARTEMIS-002 (NCT05830123)		Osteosarcoma/12 mg/kg: *n* = 10, ORR 20%	B7-H3-positive osteosarcoma	1/2025^a^
	BNT324/DB-1311		NCT05914116	I	*n* = 238, ORR 32.4%	B7-H3-positive CRPC	7/2024^b^
	Ifinatamab deruxtecan		IDeate-Pantumor01 (NCT04145622)	I/II	CRPC: *n* = 59, ORR 25%, mDOR 6.4, mPFS 4.8, mOS 13.5 ESCC: *n* = 28, ORR 21%, mDOR 3.5, mPFS 2.8, mOS 7.0 sqNSCLC: *n* = 13, ORR 31%, mDOR 4.1, mPFS NR, mOS NR ES-SCLC: *n* = 21, ORR 52%, mDOR 5.9, mPFS 5.8, mOS 9.9		
			IDeate-PanTumor02 (NCT06330064)	II	NA		
**IO**							
TIL^c^	Cadonilimab	Anti-PD-1/CTLA-4 bispecific antibody	LAST (NU24MH02^d^)	II	NA	Cervical cancer (China) Gastric/GEJ adenocarcinoma (China)	6/2022 9/2024

*ADC*, antibody–drug conjugate; *ALCL*, anaplastic large cell lymphoma; *ALK*, anaplastic lymphoma kinase; *B7-H3*, B7 homolog 3; *BRAF*, B-raf proto-oncogene; *CCA*: cholangiocarcinoma; *CI*, confidence interva; *CRC*, colorectal cancer; *CRPC*, castration-resistant prostate cancer; *ES-SCLC*, extensive-stage small cell lung cancer; *ESCC*, esophageal squamous cell carcinoma; *FGFR*, fibroblast growth factor receptor; *GEJ*, gastroesophageal junction; *H&E*, hematoxylin and eosin; *HER*, human epidermal growth factor receptor; *HR*, hazard ratio; *HR*, hormone receptor; *I-DXd*, ifinatamab deruxtecan; *IHC*, immunohistochemistry; *IMT*, inflammatory myofibroblastic tumors; *KRAS*, Kirsten rat sarcoma viral oncogene homolog; *LAST*, Tumor-Infiltrating Lymphocyte-Directed Anti-CTLA-4 and Anti-PD-1 Therapy for Immunotherapy-Naïve Solid Tumors; *mDOR*, median duration of response; *MET*, mesenchymal-epithelial transition factor; *METex14*, MET exon 14; *mOS*, median overall survival; *mPFS*, median progression-free survival; *NA*, not available; *NE*, not estimable; *NR*, not reached; *NRG1*, neuregulin 1; *NSCLC*, non-small cell lung cancer; *ORR*, objective response rate; *PDAC*, pancreatic ductal adenocarcinoma; *PD-1*, programmed death receptor-1; *PIK3CA*, phosphatidylinositol-4,5-bisphosphate 3-kinase catalytic subunit alpha; *ROS1*, proto-oncogene receptor tyrosine kinase 1; *sqNSCLC*, squamous non-small cell lung cancer; *T-DXd*, fam-trastuzumab deruxtecan-nxki; *TIL*, tumor-infiltrating lymphocyte; *TKI*, tyrosine kinase inhibitor

^a^Breakthrough therapy designation

^b^Fast track designation

^c^Based on H&E whole-slide image analysis

^d^Institutional registration; Clinicaltrial.gov registration in process

### Fibroblast growth factor receptor (FGFR) amplification, mutation, and rearrangement

FGFR is a transmembrane tyrosine kinase receptor that regulates tissue development, angiogenesis, and regeneration through downstream intracellular pathways, such as RAS/Raf/MEK and PI3K/AKT/mTOR [48]. Aberrant FGFR signaling promotes tumor growth, angiogenesis, and resistance to anticancer therapies [49]. Gene amplifications account for 66% of FGFR aberrations in human cancers, making them the most common, while FGFR mutations and rearrangements are less frequent (26%) across tumor types [50]. Urothelial carcinoma has the highest prevalence of FGFR alterations, followed by breast carcinoma, endometrial adenocarcinoma, ovarian carcinoma, glioma, squamous NSCLC (sqNSCLC), gastric adenocarcinoma, and cholangiocarcinoma (CCA) [51]. Given the widespread presence of FGFR alterations, FGFR is a potential pan-cancer biomarker.

There are currently three FDA-approved FGFR inhibitors: pemigatinib, erdafitinib, and futibatinib. Pemigatinib was approved in April 2020 for previously treated, unresectable locally advanced or metastatic CCA with FGFR2 rearrangements, based on phase 2 FIGHT 202 (NCT02924376). Erdafitinib received full approval in January 2024 for locally advanced or metastatic urothelial carcinoma with susceptible FGFR3 alterations (point mutations and fusions) after chemotherapy, based on the BLC2001 trial (NCT02365597). Futibatinib received accelerated approval in September 2022 for CCA with FGFR2 rearrangements based on the TAS-120–101 trial (NCT02052778).

Ongoing pan-tumor trials are investigating pemigatinib (NCT06022289, NCT06302621), erdafitinib (RAGNAR, MATCH subprotocol K2), and futibatinib (NCT04189445) across various FGFR-altered cancers. The phase II RAGNAR study (NCT04083976) has provided early tissue-agnostic signals, reporting an ORR of 30% (95% CI: 24–36), mPFS of 4.2 months (95% CI: 4.1–5.5), and mOS of 10.7 months (95% CI: 8.7–12.1) with erdafitinib across 16 solid tumor types (*n* = 217) [52]. Supporting this, MATCH subprotocol K2 showed ORR of 16% (90% CI 5.7–33.0) in 25 patients, along with mPFS of 3.6 months and mOS of 11.0 months [53]. For futibatinib, activity was modest in gastric or gastroesophageal junction (GEJ) cancers (*n* = 28), with an ORR of 17.9%, mDOR of 3.9 months, mPFS of 2.8 months, and mOS of 5.7 months [54]. These findings suggest the potential for broader FGFR-targeted therapeutic applications beyond currently approved indications.

Lirafugratinib received FDA breakthrough therapy designation in December 2024 for FGFR2-driven CCA and other solid tumors, recognizing its tumor-agnostic efficacy and durable responses based on the phase I/II ReFocus trial (NCT04526106). Among FGFRi-naïve FGFR2 rearrangement CCA patients (*n* = 25), the ORR was 52% (95% CI: 31.3–72.2), with an mDOR of 8.2 months (range: 1.9–18.6), and 77% of responders maintaining a response beyond 24 weeks [55]. In prior FGFRi-treated CCA patients (*n* = 50), the ORR was 14% (95% CI: 5.8–26.7), with an mDOR of 5.6 months (range: 1.9–7.4) and 57% maintaining a response beyond 24 weeks. Among FGFRi-naïve non-CCA patients (*n* = 45), the ORR was 37.8%, with an mDOR of 11.5 months (95% CI: 3.7–NE) across eight tumor types, including pancreatic (5/12), NSCLC (3/4), gastric (2/7), ovarian (2/3), breast (1/3), colorectal (1/4), combined HCC/CCA (1/1), and occult primary (2/3) [56].

### Anaplastic lymphoma kinase (ALK) rearrangement

ALK is a critical therapeutic target, as its alterations (mutations, amplifications, and rearrangements) drive tumor development by activating oncogenic pathways, including MAPK/ERK, JAK-STAT, PI3K-Akt, and PLCγ, promoting tumor cell proliferation, survival, and progression [57]. ALK rearrangements are most commonly observed in anaplastic large cell lymphoma (ALCL) and inflammatory myofibroblastic tumors (IMTs), occurring in 50–80% and ~ 50% of cases, respectively [58]. In NSCLC, ALK fusions are present in ~ 3 to 7% of cases, making it the most well-recognized solid tumor harboring ALK rearrangements [59]. However, outside of NSCLC, ALK fusions are exceedingly rare, occurring in only ~ 0.2% of cancers [58]. Given their significance, ALK inhibitors, such as crizotinib, ceritinib, alectinib, brigatinib, and lorlatinib, have gained FDA approval for ALK-altered NSCLCs, with crizotinib also approved for ALK-altered IMTs and ALCL.

Crizotinib is FDA-approved for ALK-positive NSCLC, ALCL, and IMT. To examine ALK as a tissue-agnostic biomarker, the NCI-MATCH trial subprotocol F assessed crizotinib in rare ALK-rearranged tumors, reporting an ORR of 50% (90% CI: 9.8–90.2) among five enrolled patients, including one CR, with mPFS of 3.8 months and mOS of 4.3 months [60]. Preliminary data from the iMATRIX Alectinib phase I/II study (NCT04774718) in eight pediatric patients with ALK fusion-positive tumors (high-grade glioma (*n* = 3), IMT (*n* = 2), renal cell carcinoma (*n* = 2), and ALCL (*n* = 1)) showed responses in four patients (including one CR) and stable disease in two additional patients [61]. Several ongoing trials (NCT05770037, NCT04925609, and NCT05384626) continue to explore the broad applicability of ALK inhibitors across different cancer types, focusing on treatment resistance, brain metastases, and CNS-related side effects [62].

### Mesenchymal-epithelial transition factor (MET) amplification and point mutation

MET alterations, including point mutations, gene amplifications, and fusions, drive tumor growth, invasion, and metastasis [63]. These alterations are detected in approximately 0.69% of cancers, with the highest prevalence observed in NSCLC, glioblastoma multiforme, colorectal adenocarcinoma, esophageal adenocarcinoma, and cutaneous melanoma [59].

MET inhibitors have demonstrated clinical benefit in NSCLC with MET exon 14 (METex14) skipping alterations, leading to regular approval of capmatinib and tepotinib, while crizotinib received FDA breakthrough therapy designation for this indication. Their therapeutic potential beyond NSCLC has been investigated in multiple trials. In a phase 1 study (NCT01324479), capmatinib showed antitumor activity in MET-positive non-lung tumors, with stable disease observed in 22% of gastric cancer, 46% of hepatocellular carcinoma, and 28% of other tumors [64]. In the phase II KCSG AL19-17 trial (NCT04647838), tepotinib demonstrated an ORR of 57.6% in patients with METex14 skipping mutations or MET amplification, with a mPFS of 8.0 months (95% CI: 4.5–11.5) and mOS of 14.0 months (95% CI: 7.8–20.2) [65].

Crizotinib has been evaluated in two key trials, NCI-MATCH and AcSé, assessing its efficacy in MET-altered tumors across various histologies. In the NCI-MATCH ECOG-ACRIN trial (EAY131) subprotocols C1 and C2, crizotinib showed modest clinical activity, with ORRs of 14% (90% CI: 5.0–29.8) in MET-amplified tumors (C1, *n* = 28) and 14% (90% CI: 2.6–38.5) in MET exon 14 deletion (C2, *n* = 14); mPFS was 3.4 months (90% CI: 1.8–3.7) in C1 and 2.0 months (90% CI: 1.4–4.1) in C2, while higher MET counts (≥ 50,000) in C2 correlated with improved PFS of 8.8 months (90% CI: 2.1–NA) [66]. The AcSé program (NCT02034981), led by the French National Cancer Institute, further explored crizotinib’s efficacy across multiple tumor types, reporting ORRs of 22% (95% CI: 7–38) in MET-mutated tumors, 37% (95% CI: 10–74) in MET-amplified esogastric cancer, and 24% (95% CI: 7–40) in MET-amplified NSCLC [67]. These findings underscore the broader potential of MET-targeted therapies and reinforce the role of MET alterations as a predictive biomarker across malignancies.

### Proto-oncogene receptor tyrosine kinase 1 (ROS1) rearrangement

ROS1 alterations, particularly gene fusions, are pivotal in oncogenesis by activating RAS-RAF-MEK-ERK, PI3K-AKT-mTOR, and JAK-STAT3 signaling pathways, driving tumor cell proliferation and survival [68]. ROS alterations occur in 4.2% of all cancers, with fusions most commonly found in spitzoid neoplasms (17%), IMTs (10%), CCA (1–9%), and NSCLC (1–2%) [59]. Given their transformative potential across diverse cell lineages, ROS1 fusions serve as important biomarkers for targeted therapy.

In November 2023, the FDA approved repotrectinib for locally advanced or metastatic ROS1-positive NSCLC, marking the first approval for both tyrosine kinase inhibitor (TKI)-naïve and previously treated patients. This was based on the TRIDENT-1 trial (NCT03093116), which demonstrated an ORR of 79% (95% CI: 68–88) in TKI-naïve cases (*n* = 71) and 38% (95% CI: 25–52) in prior ROS1 TKI-treated cases (*n* = 56), with mDOR of 34.1 months (95% CI, 25.6–NR) and 14.8 months (95% CI: 7.6–NR), respectively [69]. More recently, in June 2025, taletrectinib received FDA approval for the same indication based on the TRUST-I (NCT04395677) and TRUST-II (NCT04919811) trials [70]. Among TKI-naïve patients, ORR was 90% (95% CI: 83–95) in TRUST-I and 85% (95% CI: 73–93) in TRUST-II, with ≥ 12-month DOR seen in 72% and 63% of responders, respectively. In TKI-pretreated patients, ORRs were 52% (95% CI: 39–64) and 62% (95% CI: 46–75), with ≥ 6-month DOR observed in 74% and 83% of responders, respectively. These approvals provide complementary targeted options for both newly diagnosed and pretreated ROS1-positive NSCLC.

Several trials are investigating ROS1 TKIs in non-NSCLC tumors, including the NCI-MATCH trial subprotocol G, which evaluated crizotinib in rare ROS1-rearranged tumors, showing an ORR of 25% (90% CI: 1.3–75.1) in four patients, with mPFS of 4.3 months and mOS of 6.2 months [60]. Additionally, the AcSé program reported a high ORR of 54% (95% CI: 40–70) in ROS1-translocated tumors (*n* = 37), further supporting the efficacy of crizotinib in non-lung ROS1-positive tumors [67]. The DETERMINE trial (treatment arm 03) is currently recruiting patients ≥ 12 years with ROS-1 positive solid tumors to assess entrectinib’s potential as a tissue-agnostic ROS-1 targeted therapy, expanding its current indication beyond NTRK fusion-positive tumors. Similarly, the CARE study (NCT04094610) focuses on pediatric and young adult patients with advanced malignancies harboring ALK, ROS1, and NTRK1-3 gene alterations.

### Neuregulin 1 (NRG1) fusion

The NRG1 gene, located on chromosome 8, encodes NRG1, a growth factor containing an epidermal growth factor (EGF)-like domain that activates cell growth pathways. NRG1 alterations, particularly gene fusions, drive oncogenesis by activating ErbB3, which forms heterodimers with ErbB2, EGFR, and ErbB4, triggering PI3K-AKT and MAPK signaling pathways, leading to abnormal cell proliferation [71, 72]. NRG1 fusions occur in approximately 0.2% of tumors, with the highest prevalence in NSCLC, followed by gastrointestinal tumors [73]. Patients with NRG1 fusion-positive tumors often respond poorly to standard treatments and even targeted therapies, including ALK inhibitors [74].

Currently, there are no FDA-approved targeted therapies specifically for NRG1 fusions, but treatments targeting the NRG1/ErbB signaling pathway have shown both preclinical and clinical efficacy in several tumor types [75]. The FDA recently granted accelerated approval in December 2024 for zenocutuzumab, a bispecific antibody targeting HER3-mediated NRG1 signaling, as the first therapy specifically for NRG1 fusion-positive advanced NSCLC and pancreatic ductal adenocarcinoma (PDAC). This decision was based on updated results from the phase II tissue-agnostic eNRGy study (NCT02912949), which showed an ORR of 33% (95% CI: 23–59) and mDOR of 7.4 months (95% CI: 4.0–16.6) in NSCLC, while patients with PDAC achieved an ORR of 40% (95% CI: 23–59) and DOR ranging from 3.7 to 16.6 months [76, 77].

A broader tissue-agnostic analysis of zenocutuzumab in the eNRGy study and early access program included 99 patients with NRG1 + solid tumors across multiple histologies, such as NSCLC (41 patients), PDAC (18 patients), breast cancer (5 patients), CCA (3 patients), and CRC (2 patients) [78]. Among 71 patients with measurable disease, the investigator-assessed confirmed ORR was 34% (90% CI: 25–44), with responses observed in NSCLC (35%), PDAC (39%), breast cancer (2/4 patients), and CCA (1/3 patients). Responses were rapid, occurring at the first tumor assessment in 20 of 24 responders, and remained ongoing in 13 patients. The mDOR was 9.1 months (95% CI: 5.2–12.0), with a 6-month DOR rate of 70%. These findings highlight the potential of NRG1 fusions as a tissue-agnostic biomarker and support further exploration of zenocutuzumab across a broader range of tumor types.

Several other ongoing pan-cancer trials are investigating NRG1-targeted therapies, including afatinib (NCT04410653) and seribantumab (NCT04383210), an anti-HER3 monoclonal antibody demonstrating significant anti-tumor activity in patients with NRG1 fusion-positive, ERBB-targeted therapy-naïve tumors in the phase II CRESTONE study. This study reported 36% ORR (2 CR, 6 PR), a 95% disease control rate (DCR), and an overall DOR ranging from 1.4 to 17.2 months [79].

### Phosphatidylinositol-4,5-bisphosphate 3-kinase catalytic subunit alpha (PIK3CA) mutation

Phosphatidylinositol 3-kinase (PI3K) is a heterodimer kinase that regulates cellular growth and survival by activating phosphatidylinositol 3,4,5-trisphosphate pathways. The PIK3CA gene encodes the p110α catalytic subunit of PI3K, and PIK3CA mutations are reported in various human cancers, occurring in approximately 15% of cases, with higher prevalence in gastrointestinal and breast cancers [80].

Currently, all FDA-approved PI3K inhibitors are indicated for breast cancer. Alpelisib is approved for hormone receptor (HR)-positive, HER2-negative, PIK3CA-mutated advanced, or metastatic breast cancer following progression after endocrine therapy [81]. Capivasertib, in combination with fulvestrant, is approved for HR-positive, HER2-negative, locally advanced or metastatic breast cancer harboring PIK3CA, AKT1, or PTEN alterations, following progression on at least one endocrine-based regimen in the metastatic setting or recurrence within 12 months of completing adjuvant therapy [82]. Inavolisib, in combination with palbociclib (CDK4/6 inhibitor) and fulvestrant (estrogen-receptor antagonist), is approved for endocrine-resistant, PIK3CA-mutated, HR-positive, HER2-negative breast cancer [83].

Early clinical trials have demonstrated the potential efficacy of PI3K inhibitors in PIK3CA-altered solid tumors. In a first-in-human phase Ia study (NCT01219699), alpelisib (BYL719), a PI3Kα-selective inhibitor, was evaluated in 134 patients with PIK3CA-altered advanced solid tumors [84]. The ORR was 6.0%, including one CR in endometrial cancer and PRs in cervical, breast, endometrial, colon, and rectal cancers. Stable disease was observed in 70 patients (52.2%), with a DCR of 58.2%. Additionally, a phase I/II clinical trial (NCT05768139) is evaluating STX-478 in advanced solid tumors harboring PIK3CA mutations. Preliminary results in 43 patients show an ORR of 21% and DCR of 67%, with the longest PR duration exceeding 12 months [85].

Several tissue-agnostic clinical trials are investigating PI3K inhibitors to expand their therapeutic applications beyond breast cancer: NCT04439175, NCT05490771, NCT05307705, NCT06132932, NCT01449370, NCT01449370, and NCT06417391. Notably, the MATCH Subprotocol I is investigating taselisib in solid tumors harboring PIK3CA mutations without KRAS mutations or PTEN loss, while the MATCH Subprotocol Z1F is assessing copanlisib in PIK3CA-mutated cancers with primary efficacy analysis (*n* = 25) showing ORR of 16% (90% CI: 6–33), mPFS of 3.4 months (90% CI: 1.8–6.6), and mOS of 5.9 months (90% CI: 4.9–13.7) [86]. Collectively, these trials highlight the growing interest in PI3K inhibitors as tumor-agnostic therapies, reinforcing PIK3CA as a promising biomarker for targeted treatment across a wide range of cancers.

### AKT mutation

The AKT serine/threonine kinase, also known as protein kinase B, is a crucial regulator of cell growth, survival, metabolism, and migration, playing a central role in the PI3K-Akt-mTOR signaling pathway. Dysregulation of AKT signaling is implicated in various cancers and metabolic disorders, with overactive AKT driving tumor progression and resistance to therapy [87, 88]. This has led to the development of Akt inhibitors, which are classified into ATP-competitive, allosteric, and covalent-allosteric inhibitors [89].

Capivasertib (AZD5363), an ATP-competitive pan-Akt inhibitor, received FDA approval in November 2023 in combination with fulvestrant for HR-positive, HER2-negative locally advanced, or metastatic breast cancer with alterations in PIK3CA, AKT1, or PTEN, based on the phase III CAPItello-291 trial. This first-ever FDA-approved Akt inhibitor validates the therapeutic potential of targeting the AKT pathway, paving the way for further advancements in precision oncology. Ongoing research focuses on developing isoform-specific inhibitors, improving selectivity, and identifying predictive biomarkers to enhance efficacy and minimize toxicity, ultimately advancing the field of AKT-targeted therapy.

The NCI-MATCH subprotocols EAY131-Y and EAY131-Z1K provide preliminary evidence supporting the investigation of AKT as a tissue-agnostic biomarker. In the EAY131-Y study, 35 patients with AKT1 E17K-mutated metastatic tumors from diverse histologies were treated with the pan-AKT inhibitor capivasertib, achieving an ORR of 28.6% (95% CI: 15–46), including one durable CR lasting 35.6 + months in a patient with endometrioid endometrial adenocarcinoma [90]. In EAY131-Z1K, 32 patients with AKT1 E17K-mutated tumors were treated with the pan-AKT inhibitor ipatasertib, achieving an ORR of 22% (90% CI: 11–37), mDOR of 9.9 months, and 6-month PFS rate of 44% (90% CI: 29–58) [91]. Taken together, these results suggest that AKT1 E17K mutations may serve as a predictive biomarker across multiple tumor types, supporting the potential for AKT-targeted therapies in a pan-tumor approach.

Another notable pan-tumor trial evaluating capivasertib is the phase II clinical trial (NCT03310541), which highlighted allele-specific differences in AKT activation and inhibitor sensitivity, reporting an ORR of 33% and stable disease in one of six patients with non-AKT1 E17K mutations [92]. Beyond capivasertib and ipatasertib, other AKT inhibitors under development for pan-tumor indications include miransertib (NCT01473095), BAY 1125976 (NCT01915576), an AKT1/2 inhibitor, and TAS0612 (NCT04586270), which targets RSK/AKT/S6K signaling.

### Kirsten rat sarcoma viral oncogene homolog (KRAS) G12C mutation

The KRAS gene, a member of the rat sarcoma viral oncogene (RAS) family, is one of the most frequently mutated oncogenes across various cancers, including NSCLC, PDAC, and CRC. KRAS mutations drive tumorigenesis by activating cell signaling pathways, such as RAF-MEK-ERK and PI3K-AKT-mTOR, while also promoting angiogenesis and immune evasion in the tumor microenvironment (TME). Among these mutations, the G12C variant is the most prevalent, accounting for 40% of KRAS mutations and occurring in 10–13% of NSCLC, with lower frequencies in other cancers [93].

Historically, KRAS was considered “undruggable” due to structural challenges in developing direct inhibitors, leading research to focus on downstream signaling effectors or synthetic lethality approaches, such as cyclin-dependent kinase inhibitors, with limited success. However, therapeutic advancements led to the FDA’s accelerated approval of sotorasib (2021) and adagrasib (2022) for NSCLC with KRAS G12C mutations. Beyond lung cancer, adagrasib plus cetuximab received accelerated approval in June 2024 for locally advanced or metastatic KRAS G12C-mutated CRC following prior chemotherapy, based on the phase I/II KRYSTAL-1 trial (NCT03785249). Additionally, in January 2025, sotorasib plus panitumumab was approved for metastatic KRAS G12C-mutated CRC, supported by findings from the CodeBreaK 300 trial (NCT05198934).

Growing evidence supports KRAS G12C as a promising tissue-agnostic target, with multiple inhibitors showing activity across tumor types. In the first-in-human phase I study of sotorasib, confirmed responses were observed in advanced solid tumors, with an ORR of 32% and mPFS of 6.3 months in NSCLC, and an ORR of 7% with mPFS of 4.0 months in CRC; responses were also reported in pancreatic, endometrial, appendiceal cancers, and melanoma (NCT03600883) [94]. In the KRYSTAL-1 trial, adagrasib showed an ORR of 35.1% and mPFS of 7.4 months in KRAS G12C-mutated tumors excluding NSCLC and CRC (NCT03785249) [95]. A phase I study of divarasib (GDC-6036) reported ORRs of 53% and 29%, and mPFS of 13.1 and 5.6 months in NSCLC and CRC, respectively, with activity also seen in other solid tumors (NCT04449874) [96]. Most recently, the next-generation inhibitor D3S-001 demonstrated a confirmed ORR of 73.5% in KRAS G12C inhibitor-naïve patients across tumor types, including 67% in NSCLC, 89% in CRC, and 75% in PDAC (NCT05410145), further reinforcing the tissue-transcending potential of KRAS G12C-directed therapies [97].

### HER2 mutation

Genomic profiling of human cancers has identified recurrent somatic HER2 mutations, which typically occur without concurrent amplifications [98, 99]. While HER2 amplification and overexpression are well-established mechanisms in HER2-driven cancers, activating HER2 mutations can also promote tumor growth, expanding the landscape of HER2-targeted therapies.

In August 2022, the FDA approved T-DXd for metastatic HER2-mutant NSCLC, based on results from the DESTINY-Lung02 trial (NCT04644237). Beyond NSCLC, the phase II DESTINY-PanTumor01 trial (NCT04639219) demonstrated promising activity in 102 patients with various solid tumors harboring specific activating HER2 mutations, showing an ORR of 29.4% (95% CI: 20.8–39.3) [100]. These findings suggest the potential for broadening T-DXd indications to include HER2-mutant tumors, in addition to its approval for advanced solid tumors with HER2 IHC 3 + overexpression.

The ongoing phase 2 basket study SGNTUC-019 (NCT04579380) is evaluating tucatinib, a HER2 inhibitor approved for breast cancer and CRC, in combination with trastuzumab in patients with metastatic HER2-altered solid tumors. Preliminary results from the HER2-mutant metastatic breast cancer cohort (*n* = 31) show a confirmed ORR of 41.9% (90% CI: 26.9–58.2) and mPFS of 9.5 months (90% CI,:5.4–13.8), demonstrating promising clinical activity in this heavily pretreated population [101]. Additionally, several ongoing trials (MATCH-Subprotocol B/NCT04439136, DETERMINE Arm 04/NCT05786716, Beamion PANTUMOR-1/NCT06581432, NCT05315700, NCT06083662, NCT06516926, NCT06519110, NCT05274191, NCT03810872, NCT03065387) continue to investigate HER2 mutations as actionable oncogenic drivers across multiple solid tumor types.

### HER2 low/ultralow expression

The recent approval of T-DXd in January 2025 for unresectable or metastatic HR-positive, HER2-low (IHC 1 + or IHC 2 +/ISH −) or HER2-ultralow (IHC 0 with membrane staining) breast cancer underscores the growing clinical relevance of low-level HER2 expression as a biomarker beyond traditionally defined HER2-positive (IHC 3 + or ISH +) cancers. This approval builds upon the initial FDA approval of T-DXd in August 2022, which was granted for HER2-low breast cancer based on the DESTINY-Breast04 trial (NCT03734029) and included both HR-positive and HR-negative patients.

The DESTINY-Breast06 trial (NCT04494425), a randomized, multicenter, open-label study, further supported the expanded 2025 approval by demonstrating a mPFS of 13.2 months with T-DXd versus 8.1 months with chemotherapy (HR 0.62, 95% CI: 0.52–0.75; *p* < 0.0001) in patients with HER2-low breast cancer (*n* = 713) [102]. In an exploratory analysis of the HER2-ultralow subgroup (*n* = 153), mPFS was 13.2 months in the T-DXd arm compared to 8.3 months in the chemotherapy arm (HR 0.78, 95% CI: 0.50–1.21). At the time of analysis, overall survival data remained immature, requiring further follow-up. The sequential approvals of T-DXd highlight the therapeutic potential of targeting HER2 expression at sub-amplification levels, reinforcing HER2-low as an emerging biomarker with broader clinical implications.

Ongoing research continues to investigate the tissue-agnostic potential of HER2-low expression across multiple malignancies, expanding the scope for HER2-targeted ADCs like T-DXd beyond breast cancer. Evidence suggests that HER2-low expression is not exclusive to breast cancer, as recent data reveals its presence in breast (47.1%), gastric/GEJ (34.6%), salivary gland (50.0%), lung (46.9%), endometrial (46.5%), urothelial (46.0%), and gallbladder (45.5%) cancer [103]. These findings support further clinical investigation into HER2-low/ultralow as a clinically meaningful biomarker across diverse tumor types. Notably, HERTHENA-PanTumor01 (NCT06172478) is evaluating HER3-DXd, a HER3-directed ADC with demonstrated activity in EGFR-mutant metastatic NSCLC and breast cancer, in previously treated cutaneous melanoma, HER2-negative gastric cancer, and HNSCC. Other trials exploring HER2-low/ultralow expression include NCT06822998, NCT05861895, NCT06031584, NCT05461768, NCT05872295, NCT04143711, NCT03602079, and NCT06157892.

### B7 homolog 3 (B7-H3) expression

B7-H3, also known as CD276, is a transmembrane immunoregulatory protein belonging to the B7 family of immune checkpoint molecules. It functions as a co-stimulatory molecule activating antigen-presenting cells and regulating T cell responses [104, 105]. The expression of B7-H3 contributes to an immunosuppressive microenvironment by enhancing IL-10 and TGF-β1 production while suppressing suppresses key immune effectors, such as IFN-γ, IL-2, perforin, and granzyme B [106, 107]. Additionally, B7-H3 inhibits the function of CD4 + and CD8 + T cells, NK cells, macrophages, and neutrophils, allowing tumors to evade immune surveillance [108, 109].

B7-H3 is highly expressed across various malignancies, including NSCLC, CRC, cutaneous squamous cell cancer, pancreatic cancer, and breast cancer, where it promotes tumor proliferation, metastasis, and treatment resistance [110, 111]. Its expression is inversely associated with patient survival [112]. Given its limited presence in normal tissues, B7-H3 is an appealing therapeutic target for cancer treatment.

The FDA recently granted breakthrough therapy designation to GSK5764227 (GSK’227), a novel B7-H3-targeted ADC, for two indications: relapsed or refractory extensive-stage small cell lung cancer (ES-SCLC) in December 2024 and relapsed or refractory osteosarcoma in August 2024. The ES-SCLC designation is supported by early clinical evidence from the ongoing ARTEMIS-001 trial, which is evaluating the safety, tolerability, and preliminary anti-tumor activity of GSK’227 in over 200 patients with advanced solid tumors, including ES-SCLC [113]. For osteosarcoma, the designation is based on findings from ARTEMIS-002, a phase II trial that enrolled 42 patients with relapsed or refractory osteosarcoma, where no FDA-approved treatments currently exist beyond second-line therapy [114]. Global phase I/II trials are underway to further evaluate GSK’227’s efficacy across multiple tumor types.

Another promising B7-H3 ADC, BNT324/DB-1311, received fast track designation in July 2024 for unresectable advanced or metastatic castration-resistant prostate cancer (CRPC). In a phase I/IIa trial (NCT05914116) of heavily pretreated patients with locally advanced or metastatic solid tumors, BNT324/DB-1311 achieved an unconfirmed ORR of 32.4% and a DCR of 82.4% [115, 116].

The phase I/II IDeate-Pantumor01 trial (NCT04145622) evaluated ifinatamab deruxtecan (I-DXd), a novel B7-H3-directed ADC, in heavily pretreated patients with CRPC, esophageal squamous cell carcinoma (ESCC), sqNSCLC, and SCLC, showing encouraging antitumor activity irrespective of histology [117]. Confirmed ORR was 25% (15/59) in CRPC, 21% (6/28) in ESCC, 31% (4/13) in sqNSCLC, and 52% (11/21) in SCLC; mDOR was 6.4 months (95% CI: 2.8–10.6) in CRPC, 3.5 months (95% CI: 2.4–NE) in ESCC, 4.1 months (95% CI: 2.8–NE) in sqNSCLC, and 5.9 months (95% CI: 2.8–7.5) in SCLC; mPFS was 4.8 months (95% CI: 3.9–5.9) in CRPC, 2.8 months (95% CI: 1.6–4.2) in ESCC, and 5.8 months (95% CI: 3.9–8.1) in SCLC (NR for sqNSCLC); and mOS was 13.5 months (95% CI: 10.3–16.6) in CRPC, 7.0 months (95% CI: 4.8–12.2) in ESCC, and 9.9 months (95% CI: 5.8–NE) in SCLC (NR for sqNSCLC). While B7-H3 expression was moderate to high in most patients, no clear correlation was observed between expression levels and treatment response. A pharmacodynamic biomarker analysis found no significant changes in PD-L1-positive immune cells, immune cell subsets, or inflammatory gene signatures following I-DXd treatment, suggesting that I-DXd does not exert direct immunomodulatory effects [118]. These findings warrant continued clinical investigation of I-DXd in the phase Ib/II IDeate-PanTumor02 trial (NCT06330064), which is evaluating I-DXd in recurrent or metastatic solid tumors previously treated with at least one prior systemic therapy.

Other B7-H3-targeting approaches include enoblituzumab, an investigational anti-B7-H3 monoclonal antibody, which is currently under investigation in a phase I trial (NCT02982941) for children and young adults with relapsed or refractory B7-H3-positive malignant solid tumors. T-cell redirecting B7-H3 therapies are also in development. Obrindatamab, a DART molecule targeting both B7-H3 and CD3, is being investigated in a phase I trial (NCT03406949) in combination with the PD-1 inhibitor retifanlimab, with pending efficacy results. MGC018, an anti-B7-H3 ADC, is being combined with lorigerlimab (a PD-1/CTLA-4 bispecific antibody) in a phase I/Ib trial (NCT05293496) for B7-H3-expressing cancers. A unique B7-H3/IL-10 immunocytokine, IBB0979, is also under investigation in a phase I/II trial (NCT05991583) for locally advanced or metastatic solid tumors. This agent leverages IL-10 to restore CD8 + T-cell function while selectively targeting B7-H3-expressing tumors, potentially overcoming T-cell exhaustion and resistance to checkpoint inhibitors [119]. The rapid expansion of B7-H3-targeted therapies—including ADCs, monoclonal antibodies, T-cell redirecting therapies, and immunocytokines—reflects the growing recognition of B7-H3 as a tissue-agnostic target.

### Tumor-infiltrating lymphocytes (TILs)

TILs are a diverse population of lymphocytes located within and around tumors, serving as a key component of the TME. Recognized as an important prognostic marker across various solid tumors, TILs play a central role in the anti-tumor immune response. Their significance has grown with the discovery of tertiary lymphoid structures, which are well-organized clusters of TILs that enhance immune activity against tumors. Research has demonstrated that the presence, differentiation, and spatial localization of TILs within tumors strongly influence clinical outcomes [120, 121].

TIL therapy was first investigated in 1985 for metastatic melanoma and has since been explored in other solid tumors, including CRC, NSCLC, CCA, cervical cancer, and breast cancer, achieving promising responses in several cases [122–128]. In February 2024, the FDA granted accelerated approval to lifileucel, a TIL-derived autologous T-cell therapy, for unresectable or metastatic melanoma previously treated with a PD-1 inhibitor and, if BRAF V600-positive, a BRAF inhibitor with or without a MEK inhibitor. In a pivotal trial involving 89 patients, lifileucel achieved an ORR of 31.5% (95% CI: 21.1–43.4), with a mDOR not reached (95% CI: 4.1–NR).

As immunotherapy research expanded in the early 2010 s, interest in TILs intensified. A systemic review highlighted TIL’s prognostic significance, noting that the presence of immune cells like CD3 + and CD8 + T cells within the TME correlated positively with both PFS and OS across various malignancies [129]. This evidence underscored the potential role of TILs in predicting immunotherapy outcomes, shaping treatment strategies, and guiding patient selection.

Advances in technology have facilitated enhanced characterization of TILs, including their phenotypic and functional properties, spatial distribution, and interactions with malignant and non-malignant cells. Recent studies have applied artificial intelligence (AI)-powered spatial analysis of hematoxylin and eosin (H&E)-stained whole-slide images (WSI) to assess TIL density and immune phenotypes (IPs) as a potential biomarker for predicting tumor response to ICIs in multiple cancers. In a NSCLC cohort from The Cancer Genome Atlas, an AI-based model was used to visualize immune phenotypes and analyze gene expression and mutational profiles [130]. Based on TIL density, the TME was classified into three IPs: inflamed (high TIL infiltration), immune-excluded (TILs restricted to the periphery of the tumor), and immune-desert (low TIL presence). The inflamed IP consistently exhibited the highest cytolytic scores, immune cell infiltration, and hallmark immune pathway activation. A follow-up study using H&E-stained WSIs from NSCLC patients further validated these findings. The inflamed IP correlated with higher immune cytolytic activity, increased response to ICI therapy, and longer mPFS and mOS compared to other IPs [131].

Expanding this AI-based IP classification across multiple tumor types, a multicenter retrospective analysis of H&E WSIs from 1806 ICI-treated patients across 27 solid tumors demonstrated significantly improved outcomes in the inflamed IP [132]. In this study, the inflamed IP was present in 35.2% of cases. Compared to patients with non-immune IP, those with inflamed IP had an ORR of 26.3% vs. 15.8%, mPFS of 5.3 vs. 3.1 months (HR 0.68, 95% CI: 0.61–0.76), and mOS of 25.3 vs. 13.6 months (HR 0.66, 95% CI: 0.57–0.75) (*p* < 0.001 for all comparisons). Subgroup analyses confirmed the prognostic value of IIP across major patient groups (ICI regimen, treatment line, PD-L1 tumor proportion score status, TMB status, histologic subtype, specimen type, tissue harvest site, timing of tissue collection) except for those with MSI-H/dMMR. These findings suggest that AI-based inflamed IP could serve as a practical and tumor-agnostic biomarker for guiding ICI therapy. Furthermore, using AI to analyze routine H&E slides enables a time-efficient, labor-efficient, and objective approach, minimizing interobserver variability and improving reproducibility.

Several clinical trials are investigating TILs as a biomarker to optimize treatment strategies in multiple tumor types. In triple-negative breast cancer, ongoing studies are exploring chemotherapy de-escalation (NeoTRACT/NCT05645380, OPTImaL/NCT06476119, ETNA/NCT06078384, NCT05929768) or exploring ICI-only neoadjuvant regimens (BELLINI/NCT03815890, POP-DURVA/NCT05215106, NEOASIS/NCT06279130). In breast cancer of various subtypes, the NCT05206396 trial is examining the relationship between TIL density and pathologic CR after neoadjuvant therapy. Meanwhile, the NCT06763640 trial is a multicenter study exploring the prognostic significance of TIL infiltration in nasopharyngeal carcinoma using deep learning-based digital pathology analysis to guide personalized treatment strategies. Collectively, these trials highlight the growing interest in TILs as a predictive biomarker for treatment adaptation.

Beyond individual tumor types, the Tumor-Infiltrating Lymphocyte-Directed Anti-CTLA-4 and Anti-PD-1 Therapy for Immunotherapy-Naïve Solid Tumors (LAST trial) will be the first pan-tumor, TIL-based trial, evaluating cadonilimab, a bispecific anti-CTLA-4/PD-1 ICI, in patients with inflamed immune phenotypes. This multi-center study aims to assess the efficacy of TIL-directed immunotherapy across diverse solid tumors, further solidifying TILs as a tumor-agnostic predictive biomarker for immunotherapy response.

## Future directions

As tissue-agnostic biomarkers broaden therapeutic opportunities across histologies, their success hinges on a nuanced understanding of tumor-specific biology. This is notably evident in BRAF V600E mutations, where response to treatment varies by tumor type—requiring monotherapy in some cancers, a combination with MEK inhibitors in melanoma, and a combination with EGFR inhibitors in CRC to counteract feedback activation pathways and inherent resistance [133–136]. Similarly, the predominance of classical activating EGFR mutations in lung cancer highlights the role of tissue-specific molecular aberrations in dictating therapeutic responses [137]. NTRK gene fusions, while actionable across multiple tumor types, are disproportionately prevalent in cancers like mammary analogue secretory carcinoma and secretory breast carcinoma, suggesting a potential lineage-dependent context that influences response to targeted therapy [9].

TMB, although FDA-approved as a predictive biomarker for PD-1 blockade, exemplifies the challenges of real-world translation due to its variable threshold reliability across tumor types, inconsistent tissue-versus-blood concordance, and potential sampling bias in heterogeneous tumors [138–141]. Moreover, real-world implementation lags significantly, with studies showing that only a fraction of eligible patients with rare biomarker-defined cancers actually receive the corresponding targeted therapy [142].

Given the heterogeneity of tumor biology, single-biomarker approaches may fall short in capturing the complexity of certain malignancies, particularly cancers of unknown primary or rare, understudied tumors. Future research should prioritize multiomic integration—including genomic, transcriptomic, proteomic, metabolomic, radiomic, and clinical data—to better understand how tumor-agnostic biomarkers interact with lineage-specific pathways. Emerging frameworks like synthetic lethality, pan-essentiality, and response taxonomies (e.g., tumor-agnostic vs. tumor-modulated vs. tumor-restricted) offer conceptual models for tailoring intervention strategies [143].

The ETAC-S tool, which proposes ≥ 20% ORR in at least four tumor types with ≥ 5 evaluable patients per type, represents a pragmatic benchmark for agnostic activity, but likely requires ongoing refinement as more real-world and trial data accumulate [144]. Meanwhile, adaptive clinical trials—basket, platform, and N-of-1 designs—can enable more responsive evaluation of cross-histology therapies. Embedding real-world evidence and early regulatory engagement into these trials will be crucial to accelerate access for patients without histology-specific options.

## Conclusions

Currently, six tissue-agnostic biomarkers and nine corresponding therapies are FDA-approved. While they offer critical avenues for patients with limited options, their optimal deployment demands integration of biomarker data with tumor lineage, immune phenotype, and resistance mechanisms. Early molecular testing should be foundational to all diagnostic workups, and new predictive markers, such as TILs, should be rigorously evaluated across tumor types, especially in immunotherapy. Ultimately, bridging tumor-agnostic strategies with lineage-aware precision will be the next frontier in oncology. By coupling multiomic profiling with adaptive trial design and equitable implementation, the field can deliver on the promise of personalized cancer care—regardless of histologic origin.

## Data Availability

No datasets were generated or analysed during the current study.

## Abbreviations

ADC: Antibody-drug conjugate
AI: Artificial intelligence
AKT: Protein kinase B
ALCL: Anaplastic large cell lymphoma
ALK: Anaplastic lymphoma kinase
APC: Antigen-presenting cell
B7-H3: B7 homolog 3
BRAF: B-raf proto-oncogene
CCA: Cholangiocarcinoma
CI: Confidence interval
CR: Complete response
CRC: Colorectal cancer
CRPC: Castration-resistant prostate cancer
CTLA-4: Cytotoxic T-lymphocyte-associated protein 4
DCR: Disease control rate
DNA: Deoxyribonucleic acid
dMMR: Mismatch-repair deficiency
EGF: Epidermal growth factor
EGFR: Epidermal growth factor receptor
ERK: Extracellular signal-regulated kinase
ES-SCLC: Extensive-stage small cell lung cancer
ESCC: Esophageal squamous cell carcinoma
ESMO: European Society for Medical Oncology
FDA: Food and Drug Administration
FGFR: Fibroblast growth factor receptor
FISH: Fluorescence *in situ* hybridization
GDNF: Glial cell-derived neurotrophic factor
GEJ: Gastroesophageal junction
GFRα: Glial cell-derived neurotrophic factor family receptor-α
GzmB: Granzyme B
HER: Human epidermal growth factor receptor
HGG: High-grade glioma
HR: Hazard ratio
HR: Hormone receptor
H&E: Hematoxylin and eosin
ICI: Immune checkpoint inhibitors
I-DXd: Ifinatamab deruxtecan
IFNγ: Interferon-gamma
IHC: Immunohistochemistry
IMT: Inflammatory myofibroblastic tumors
IP: Immune phenotype
ISH: *In situ* Hybridization
LGG: Low-grade glioma
KRAS: Kirsten rat sarcoma viral oncogene homolog
LAST: Tumor-infiltrating lymphocyte-directed anti-CTLA-4 and anti-PD-1 therapy for immunotherapy-naïve solid tumors
MAPK: Mitogen-activated protein kinase
mDOR: Median duration of response
MEK: Mitogen-activated protein kinase kinase
MET: Mesenchymal-epithelial transition factor
METex14: MET exon 14
MMR: Mismatch-repair
mOS: Median overall survival
mPFS: Median progression-free survival
MSI: Microsatellite instability
MSI-H: Microsatellite instability-high
mTOR: Mechanistic target of rapamycin
NA: Not available
NCCN: National Comprehensive Cancer Network
NE: Not estimable
NR: Not reached
NRG1: Neuregulin 1
NSCLC: Non-small cell lung cancer
NTRK: Neurotrophic tyrosine receptor kinases
ORR: Objective response rate
OS: Overall survival
PDAC: Pancreatic ductal adenocarcinoma
PD-1: Programmed death receptor-1
PD-L1: Programmed death receptor ligand-1
PFS: Progression-free survival
PFN: Perforin
PI3K: Phosphoinositide 3-kinase
PIK3CA: Phosphatidylinositol-4,5-bisphosphate 3-kinase catalytic subunit alpha
PR: Partial response
RET: Rearranged during transfection
RAF: Rapidly accelerated fibrosarcoma
ROS1: Proto-oncogene receptor tyrosine kinase 1
sqNSCLC: Squamous non-small cell lung cancer
T-DXd: Fam-trastuzumab deruxtecan-nxki
TIL: Tumor-infiltrating lymphocyte
TKI: Tyrosine kinase inhibitor
TMB: Tumor mutation burden
TMB-H: Tumor mutation burden-high
TME: Tumor microenvironment
TNFα: Tumor necrosis factor-alpha
TRK: Tropomyosin receptor kinase
WSI: Whole-slide images
